# Transcriptomic Analysis of Distal Parts of Roots Reveals Potentially Important Mechanisms Contributing to Limited Flooding Tolerance of Canola (*Brassica napus*) Plants

**DOI:** 10.3390/ijms232415469

**Published:** 2022-12-07

**Authors:** Mengmeng Liu, Janusz J. Zwiazek

**Affiliations:** 1 College of Agriculture, Guizhou University, Guiyang 550025, China; 2 Department of Renewable Resources, University of Alberta, Edmonton, AB T6G 2E3, Canada

**Keywords:** roots, hypoxia, aquaporins, transcription factors

## Abstract

Since most of the root metabolic activities as well as root elongation and the uptake of water and mineral nutrients take place in the distal parts of roots, we aimed to gain insight into the physiological and transcriptional changes induced by root hypoxia in the distal parts of roots in canola (*Brassica napus*) plants, which are relatively sensitive to flooding conditions. Plants were subject to three days of root hypoxia via lowering oxygen content in hydroponic medium, and various physiological and anatomical features were examined to characterize plant responses. Untargeted transcriptomic profiling approaches were also applied to investigate changes in gene expression that took place in the distal root tissues in response to hypoxia. Plants responded to three days of root hypoxia by reducing growth and gas exchange rates. These changes were accompanied by decreases in leaf water potential (*Ψ*_leaf_) and root hydraulic conductivity (*L*_pr_). Increased deposition of lignin and suberin was also observed in the root tissues of hypoxic plants. The transcriptomic data demonstrated that the effect of hypoxia on plant water relations involved downregulation of most *BnPIP*s in the root tissues with the exception of *BnPIP1*;*3* and *BnPIP2*;*7*, which were upregulated. Since some members of the PIP1 subfamily of aquaporins are known to transport oxygen, the increase in *BnPIP1*;*3* may represent an important hypoxia tolerance strategy in plants. The results also demonstrated substantial rearrangements of different signaling pathways and transcription factors (TFs), which resulted in alterations of genes involved in the regulation of *L*_pr_, TCA (tricarboxylic acid) cycle-related enzymes, antioxidant enzymes, and cell wall modifications. An integration of these data enabled us to draft a comprehensive model of the molecular pathways involved in the responses of distal parts of roots in *B. napus*. The model highlights systematic transcriptomic reprogramming aimed at explaining the relative sensitivity of *Brassica napus* to root hypoxia.

## 1. Introduction

Canola (*Brassica napus*) is an economically important crop plant that is widely cultivated for oil production [1]. In Canada, *B. napus* is seeded shortly after the snowmelt in spring, which leads to high risks of flooding events reducing oxygen supply to roots [2]. Since *B. napus* plants are relatively sensitive to root hypoxia [3,4], even short-term flooding events can cause high mortality and severely reduce growth and seed production of surviving plants [5]. Diminished oxygen supply alters aerobic metabolism and morphological and physiological traits in roots and triggers a chain of events that affect morphological and physiological traits in plants, including gas exchange, nutrient uptake, and water relations [6,7].

Effects of root hypoxia on plant water relations can be largely explained by decreased root water uptake and transport [4,8,9]. Impeded water delivery to shoots due to root hypoxia has been attributed to the effects on the function of root aquaporins as well as alterations of root structure, including the deposition of suberin and lignin [4], which decrease root hydraulic conductivity [10]. In addition to water, aquaporins are involved in transporting other small molecules across cell membranes, including CO_2_ [11], O_2_ [12], H_2_O_2_ [13], lactic acid [14], and some ions [15], which may affect plant responses to hypoxia. Transport of these molecules could help plants survive short exposures to flooding. For example, transport of lactic acid through NIP2;1 under hypoxic conditions could alleviate hypoxia stress by preventing its accumulation in root cells [14]. In *Nicotiana tabacum*, NtPIP1;3 was shown to help plants survive flooding by enhancing oxygen delivery to roots [12]. Although the alignment of NtPIP1;3 showed an overall greater similarity to BnPIP1;1 and BnPIP1;2 compared with BnPIP1;3, the molecular basis of oxygen transport through aquaporins remains unknown and it is possible that BnPIP1;3 may also act as an oxyporin. Transport regulation of these molecules across cell membranes may involve changes in transcript abundance of aquaporins, their post-translational modifications, membrane trafficking [16], and channel gating [17]. Water channel closure may be triggered by hypoxia through a reduction of cytosolic pH and depletion of ATP that may be required for aquaporin phosphorylation [18].

Plants have evolved different signaling pathways in response to hypoxia and other environmental stresses [19]. The activation of abscisic acid (ABA), Ca^2+^, reactive oxygen species (ROS), and the ethylene signaling pathway induces various defense responses [20,21]. Calcium and ABA are closely associated with the activation of glycolytic enzymes [22] and transcription factors, including WRKY, under oxygen-limiting condition [23]. Ethylene signaling is involved in forming adventitious roots [24] and enhancing water transport across the aquaporins [8,25], while ROS are associated with a formation of aerenchyma [26].

Hypoxia may affect multiple key functions in the distal parts of plant roots since they are involved in the uptake of water and nutrients and are the site of synthesis of various metabolites and hormones that are essential to plant survival [4,5]. However, relatively few studies have focused on the functional traits of these important parts of roots in response to hypoxia. Genes encoding transcription factors (TFs), which repress or activate the expression of their target genes [27], are also essential for the initiation of a series of stress responses that may include modification of root structure [28]. TFs that belong to the WRKY, mitogen-activated protein kinase (MAPK) cascade, basic helix–loop–helix (bHLH)-type, MYB, AP2-ERF, and NAC families have been identified to be critical in plant responses to hypoxia stress [29]. AP2-ERF TFs were upregulated under low oxygen conditions in *Arabidopsis*, rice, and poplar (*Populus trichocarpa*) [30,31] and shown to regulate genes encoding respiratory enzymes and ROS scavengers [32]. In addition, some of the TFs may be involved in regulation of aquaporin expression, including MYB [33] and ERF [34]. Translucent green, which belongs to the ERF family, was reported to directly regulate the expression of *AtTIP1*;*1*, *AtTIP2*;*3*, and *AtPIP2*;*2* in *Arabidopsis* [34], and *OsPIP2*;*2* in *Oryza sativa* triggers translocation of OsmaMYB TF [33].

RNA-Seq has been increasingly applied to rapidly analyze mRNA profiles in a wide range of plants exposed to adverse environmental conditions. The “reverse genetics” strategy helps identify candidate genes for plant improvement and for a better understanding of stress resistance mechanisms. Recently, transcriptome analysis was carried out with *Brassica napus* roots [3,35] and shoots [36] to examine the responses of plants to waterlogging. However, transcriptomic rearrangements in newly formed distal parts of roots have not yet been adequately characterized. Therefore, our study focused on changes in the distal root regions in response to root hypoxia to explain, through a comprehensive transcriptomic study of the root distal parts, the mechanisms which may contribute to the limited tolerance of root hypoxia by *B. napus* plants.

In the present study, we hypothesized that aquaporins in the distal parts of roots in canola (*Brassica napus*) are the key factors in determining water balance and TFs are essential for the responses to hypoxia stress. To verify this hypothesis, *B. napus* seedlings were exposed for three days to root hypoxia treatments, and plant physiological responses and transcriptional regulatory pathways of the distal root regions were investigated. The objective of this study was to provide a better insight into the responses of the distal root regions, which are key to water and nutrient uptake, and to identify potential key regulators of plant responses to root hypoxia.

## 2. Results

### 2.1. Physiological and Anatomical Responses of Plants to Hypoxia

**Figure 1:**
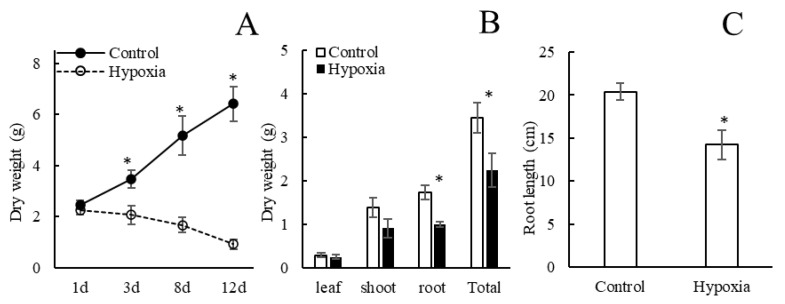
Total plant dry weight change of different hypoxia treatment durations (**A**), dry weight of different part of plants (**B**) and root length (**C**) of control plants and in plants subjected to hypoxia for three days. Means ± SE are shown (*n* = 6–8). Asterisks indicate a difference between aerated and hypoxia (*p* ≤ 0.05 denoted by *; *t*-test).

To determine the best time points for transcriptome sequencing, changes in root dry weights were measured after different hypoxia durations. A significant reduction of the total plant dry weight compared with aerated control occurred after three days of the hypoxia treatment (Figure 1A). Root dry weights in hypoxic plants significantly decreased compared with control plants, while leaf and shoot dry weights showed no significant (*p* ≤ 0.05) change (Figure 1B). Root lengths were markedly lower in hypoxic plants compared with the well-aerated control (Figure 1C).

**Figure 2:**
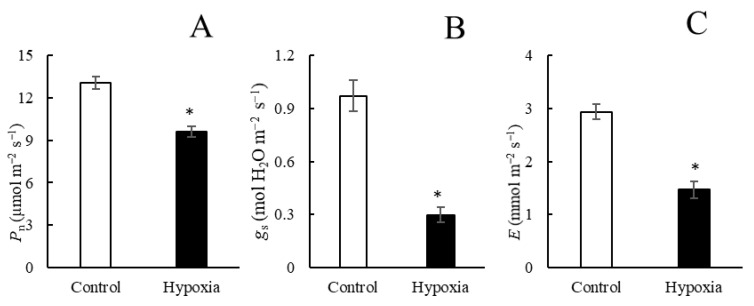
Net photosynthesis (*P*_n_, (**A**)), stomatal conductance (*g*_s_, (**B**_), and transpiration rate (*E*, (**C**)) in control plants and in plants subjected to hypoxia for three days. Means ± SE are shown (*n* = 6–8). Asterisks indicate statistically significant differences between aerated and hypoxia (*p* ≤ 0.05 denoted by *; *t*-test).

**Figure 3:**
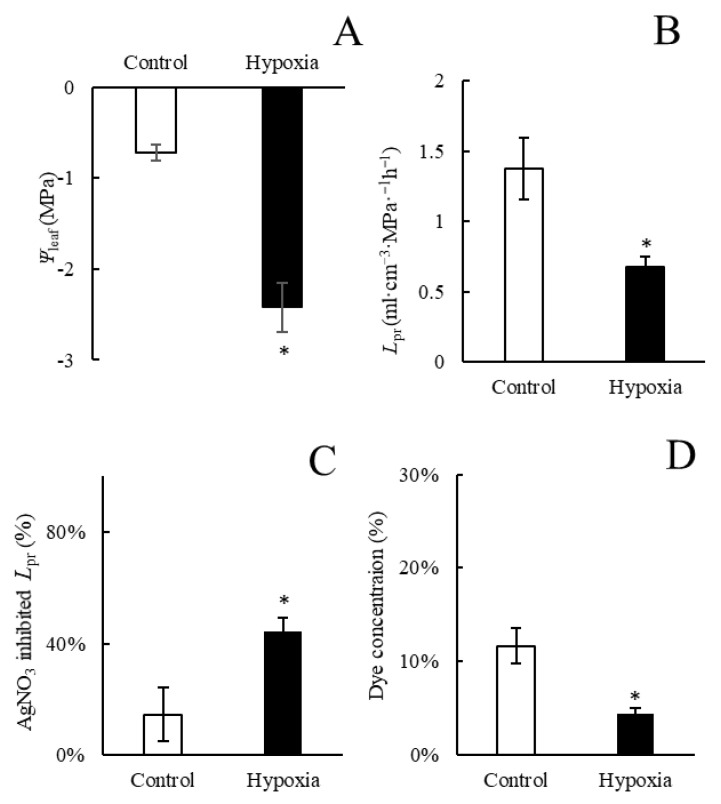
Leaf water potential (*Ψ*_leaf_, (**A**)),root hydraulic conductivity (*L*_pr_, (**B**)), *L*_pr_ in plants treated with AgNO_3_ (% control) (**C**), and percentage of the apoplastic light green SF yellowish dye concentration of the concentration applied to roots (**D**) in aerated plants and in plants subjected to hypoxia for three days. Means ± SE are shown (*n* = 6–8). Asterisks indicate a difference between aerated and hypoxia (*p* ≤ 0.05 denoted by *; *t*-test).

Gas exchange displayed similar trends as the growth traits. Net photosynthesis rate (*P*_n_), stomatal conductance (*g*_s_), and transpiration rates (*E*) were reduced by 26.3%, 69.3%, and 50%, respectively, after three days of the hypoxia treatment (Figure 2A–C). Leaf water potential (*Ψ*_leaf_) markedly decreased after three days of hypoxia (Figure 3A). Similarly, root hydraulic conductivity (*L*_pr_) was significantly lower after three days of hypoxia treatment compared with the control plants (Figure 3B). In addition, the aquaporin inhibitor AgNO_3_ was used to detect the aquaporin-mediated *L*_pr._ The results indicated that AgNO_3_ inhibited *L*_pr_ in hypoxic roots significantly more compared with control roots. Apoplastic dye solution was used to detect the apoplastic percentage of *L*_pr_, and the results showed that the proportion of apoplastic *L*_pr_ in control roots was markedly higher compared with hypoxic roots.

**Figure 4:**
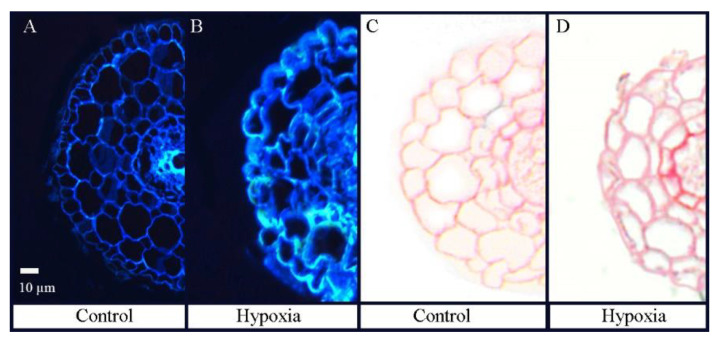
Cross sections of distal root segments in control *B. napus* plants (**A**,**C**) and in plants treated for three days with root hypoxia (**B**,**D**). Lignin autofluorescence was visualized following UV irradiation (**A**,**B**). Suberin deposition stained with Sudan 7B was visualized with light microscopy (**C**,**D**).

Lignin and suberin deposition in distal root segments was observed under the microscope. More intensive lignin autofluorescence of the cell walls in hypoxic distal root segments was observed compared with control plants (Figure 4A,B). Furthermore, increased suberin deposition was detected in the endodermis of the hypoxic roots (Figure 4C,D).

### 2.2. Mapping and Differential Gene Expression Analysis

The RNA samples from the root segments of aerated *B. napus* and hypoxic plants were collected for sequencing. Approximately 48.5 million reads were generated from each sample (Table 1). Approximately 77.32% of the reads were mapped to the B. napus reference genome and ∼2.5 to 3 million reads were mapped to multiple regions (Table 1).

### 2.3. Overall Identification and Functional Annotation of Differentially Expressed Genes

**Figure 5:**
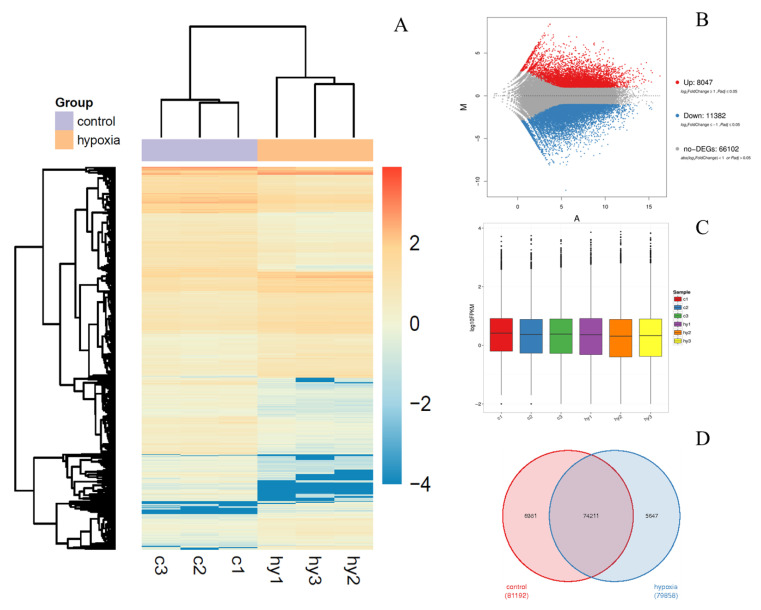
Expression analysis of the differentially expressed genes (DEGs) in distal root tissues of three samples of aerated control plants (c1, c2, c3) and in three samples from plants subjected to three days of root hypoxia (hy1, hy2, hy3). (**A**) Hierarchical cluster analysis with different columns in the figure representing different samples, and different rows representing different genes. The colors from blue to yellow indicate gene expression from low to high respectively. (**B**) Bland–Altman (MA) plot. Each point in the MA plot represents a gene. The blue points represent downregulated genes, the red points represent upregulated genes, and the grey points represent unchanged genes. (**C**) Fragments per kilobase of transcript per million mapped reads (FPKM) boxplot. (**D**) Venn diagram representing the specific and common DEGs across the control vs. hypoxia.

To obtain a general overview of differently expressed genes in *B. napus* root distal segments in response to hypoxia treatment, the expression analyses were carried out with three aerated and three hypoxic distal root segments. The hierarchical cluster (H-cluster) analysis of all DEGs (differently expressed genes) is shown in Figure 5A. The overall distribution of the gene expression abundances and differential fold changes in the two groups are shown in the MA plot (Figure 5B). The total number of differentially expressed genes (DEGs) between two groups was 19,429. There were 8047 upregulated and 11,382 downregulated DEGs (Figure 5B). The expression level in each sample was measured via the overall discrete level of expression (Figure 5C). The protein encoding gene expression level FPKM (fragments per kilobase million) had values across 10^−2^ to 10^4^ orders of magnitude. The dispersion degree of sample gene expression level distribution was average (Figure 5C). The Venn diagram revealed a total of 74,211 common genes were found in both control and hypoxic root distal segments (Figure 5D).

**Figure 6:**
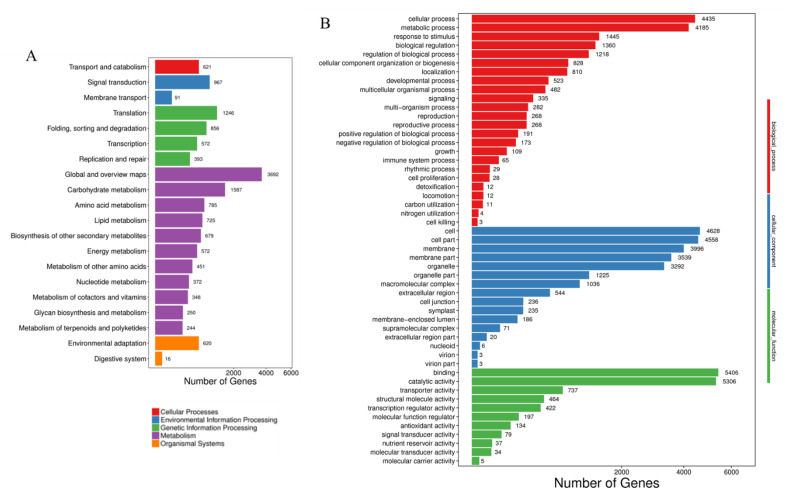
Classification of *B. napus* hypoxia stress responsive genes for each KEGG category. (**A**) Kyoto Encyclopedia of Genes and Genomes (KEGG) pathway enrichment of DEGs in *B. napus*. The x-axis shows the number of genes. (**B**) Gene ontology (GO) analyses of the DEGs. The y-axis shows the names of the GO terms, and the x-axis presents the number of genes.

To identify active biochemical pathways in response to hypoxia stress, the KEGG (Kyoto Encyclopedia of Genes and Genomes) database was used to classify and characterize the DEGs into corresponding pathways. Based on the abundantly enriched DEG numbers, the top 20 pathways are listed. The most abundant DEGs included global and overview maps (3692) in metabolism, translation (1246) in genetic information processing, signal transduction (967) in environmental information processing, transport and catabolism (621) in the cellular process, and environmental adaption (620) in organism systems (Figure 6A).

The GO enrichment analysis was performed to obtain functional information for the DEGs. Significantly enriched GO terms, identified based on FDR ≤ 0.01, were found for DEGs involved in biological processes, cellular components, and molecular functions (Figure 6B). The DEGs found in hypoxic *B. napus* were mainly enriched in the cellular (4435) and metabolic process (4185) in biological processes. In the cellular component category, the highly represented dominant subcategories were cell (4628) and cell part (4558). Among molecular function terms, binding (5406) and catalytic activity (5306) were the most highly represented (Figure 6B).

### 2.4. Validation of the Differentially Expressed Genes (DEGs) via qRT-PCR

**Figure 7:**
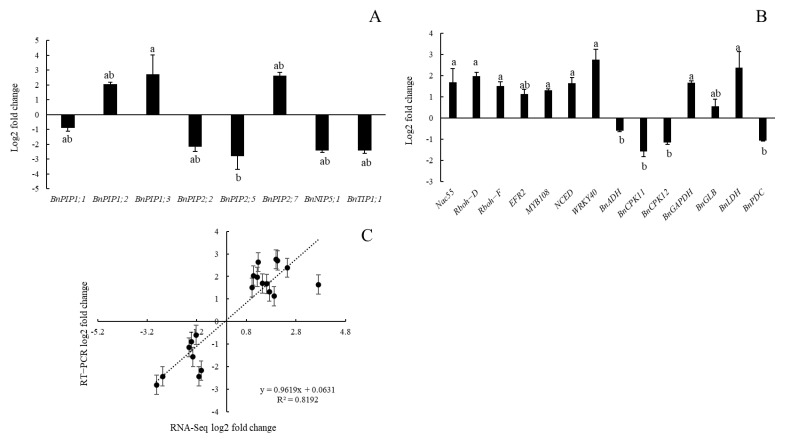
Validation of RNA-seq results through qRT-PCR analysis. (**A**,**B**) Fold change of differentially expressed genes through qPCR method. (**C**) Correlation analysis of differentially expressed genes between qPCR analysis and RNA-seq experiment. Different letters indicate statistically significant differences between different genes (Tukey’s test; *p* ≤ 0.05; one-way ANOVA).

To verify the reliability and accuracy of the Illumina RNA seq data, quantitative real-time reverse transcription-PCR (qRT-PCR) was used to assess the relative expression levels for 22 key genes closely related to hypoxia response. This independent evaluation revealed the reliability of the RNA-seq data. Figure 7A shows that the expression of aquaporin genes, including PIPs, NIPs, and TIPs, was mostly downregulated in the distal root segments in response to hypoxia stress except for PIP1;2, PIP1;3, and PIP2;7 (Figure 7A). Genes encoding signals such as NAC55, Rbohs, and NCED, as well as crucial transcription factors ERF2 and WRKY40, were significantly upregulated under hypoxia (Figure 7B). The correlation between qRT-PCR and RNA-seq was measured via scatter plotting log2-fold changes, which showed a positive correlation coefficient in both techniques (R^2^ = 0.8192) (Figure 7C).

### 2.5. DEGs Encoding Transcription Factors (TFs)

**Figure 8:**
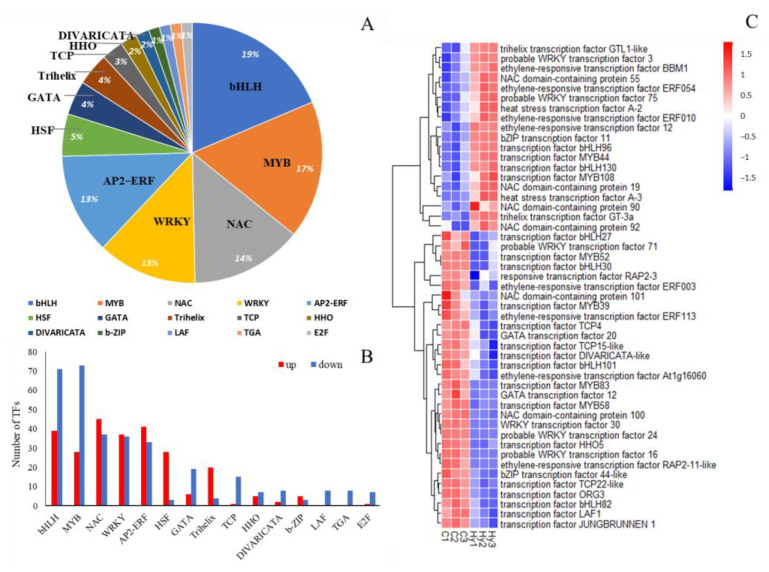
The analysis of differentially expressed transcription factors (TFs) in 3 days of hypoxia-treated roots. (**A**) The pie chart presents the percentage of 15 different families’ categories of transcription factors. TFs less than 1% of the total are not marked. Differentially expressed TFs in drought stress. (**B**) The distribution of transcription factors in upregulated and downregulated TF families. (**C**) The heatmap transcript profiles of selected transcription factor genes related to hypoxia response in *B. napus* roots. Columns and rows in the heatmap represent samples and genes, respectively. Sample names are displayed below the heatmaps. The color bar is the scale for the expression levels of each gene.

To identify the TFs involved in the regulation of plant response to hypoxia stress, 457 *B. napus* TFs distributed in 19 families were identified. Hypoxia stress induced or suppressed many TFs in hypoxic B. napus root distal segments. The top six most abundant TF families were bHLH, MYB, NAC, WRKY, AP2-ERF, and HSF (Figure 8A). Specifically, NAC and AP2/ERF were the TF families with the largest number of upregulated DEGs, while bHLH and MYB families had most of the downregulated DEGs caused by hypoxia stress (Figure 8B). Among the detected TFs, most of the NAC and ERF TF families displayed marked upregulation in the hypoxic distal root segments (Figure 8C).

### 2.6. DEGs Encoding Hypoxia Responsive Genes

**Figure 9:**
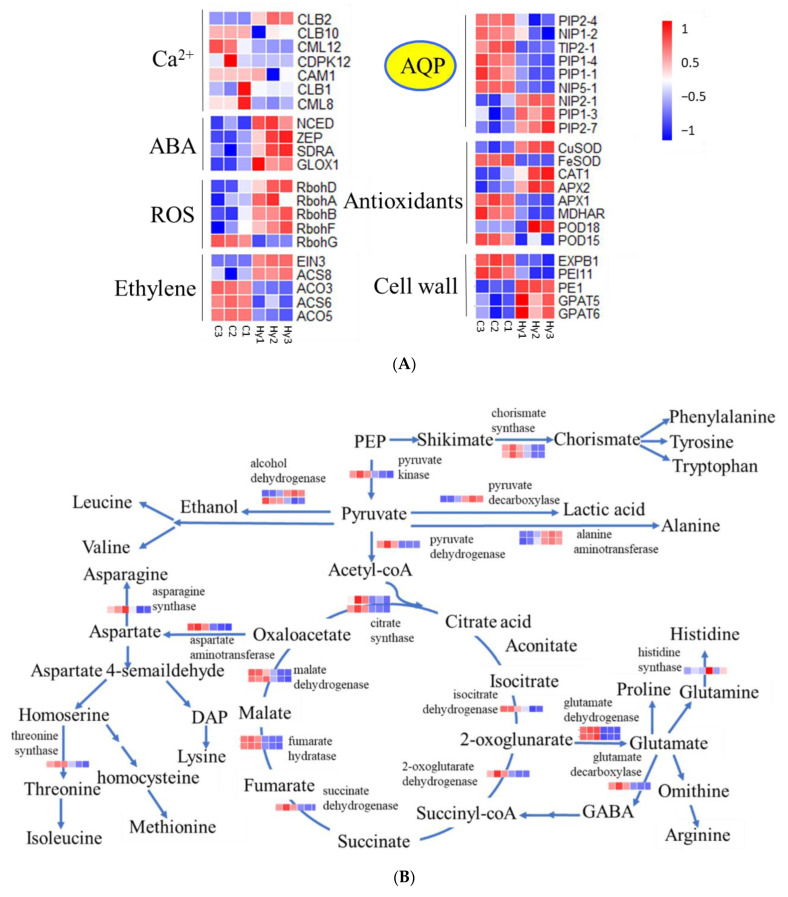
A model of hypoxia-response mechanisms in *B. napus*. (**A**) Hypoxia stress in roots is rapidly recognized through ABA, ROS, and ethylene signaling pathway. The signaling pathways stimulate a transcriptional cascade. The physiological-responsive genes are associated with a range of metabolic activities including cell wall composition adjustments, AQP alterations, antioxidants production, and TCA related enzymes. (**B**) Hypoxia-induced genes involved in respiration processes. Genes were labeled using individual heatmaps. The color bar is the scale for the expression levels of each gene on the basis of FPKM (fragments per kilobase million) value.

According to transcriptome gene expression heatmaps, Ca^2+^, ABA, ROS and the ethylene signaling pathway were altered in response to the hypoxia treatment (Figure 9A). In addition, signal transduction of TFs resulted in downstream physiological responses such as cell wall modification, decreased *L*_pr_, increased antioxidant systems, and ATP shortage crisis (Figure 9A). We further determined gene-encoded enzymes associated with the TCA cycle, which were also enriched among the DEGs (Figure 9B). In the TCA cycle, the expression of the majority of DEGs was downregulated. These results were consistent with the results of physiological measurements and root anatomy observations.

## 3. Discussion

Root hypoxia is one of the common environmental factors limiting plant growth. Root is the first organ confronted with hypoxia stress during flooding events and the newly formed roots must retain their functionality for plant survival. The effects of hypoxia on growth and physiological processes have been well documented in many plant species including *B. napus* [37]. However, few studies have focused on transcriptomic responses in root tips and the mechanisms contributing to limited hypoxia tolerance of waterlogging-sensitive species.

In the present study, three days of hypoxia stress had profound effects on the physiological parameters and affected plant dry weights. We found no adventitious roots or aerenchyma in *B. napus* plants subjected to hypoxia, and our study results are consistent with previous research, which showed that *B. napus* plants are sensitive to root hypoxia [4]. Furthermore, similarly to studies with other plants [16,38], we demonstrated that gas exchange and plant water relations in *B. napus* were significantly inhibited compared with well-aerated plants and hypoxic roots accumulated significant amounts of lignin and suberin. These plant responses point to root water transport as one of the processes in *B. napus* that are sensitive to hypoxia.

Comparative analyses of the RNA-Seq data from the hypoxic *B. napus* distal root tissues revealed that a total of 19,429 genes were differentially expressed. The KEGG analysis indicated that more DEGs were involved in the metabolic pathway, biosynthesis of secondary metabolic pathways, plant hormone and transduction pathway. GO analysis showed most DEGs participate in the metabolic process. Therefore, we mainly focused on the transcriptomic responses of aquaporins, TF responses, signaling pathways, and related physiological and morphological changes and explained the reason for the sensitivity of *B. napus* to hypoxia.

### 3.1. Aquaporin Expression

Due to the importance of aquaporins in transporting water and other small molecules including gases, lactic acid, and some ions, maintaining their functionality is one of the key elements of hypoxia tolerance [17]. In our study, most of the PIPs that may be responsible for water transport [10] were downregulated. In addition, *TIP1*;*1* and *TIP2*;*1*, which were reported as potential water transporters [39], were downregulated in response to the root hypoxia treatment. The results were consistent with the waterlogging-sensitive genotype of sorghum [39] and explain the reduction of *L*_pr_ and *Ψ*_leaf_. Interestingly, *PIP1*;*3* and *PIP2*;*7* were upregulated upon hypoxia. Previous studies indicated that tobacco PIP1;3 is involved in transporting oxygen under hypoxia [12], which could explain the reason for its upregulation. A possible function of PIP2;7 under hypoxia is unclear. Most of the BnPIP2s are water transporters [40], but their functionality may be differently regulated and differently affected by stress factors. Therefore, it could be speculated that the upregulation of PIP2;7 could represent an attempt by hypoxic plants to restore water homeostasis. Additionally, in our study, *NIP2*;*1* and other *NIP*s were upregulated, which may suggest that, similarly to NIP2;1 in *Arabidopsis* [14], NIPs in *B. napus* may be involved in the efflux of lactic acid to reduce its cell accumulation during hypoxia.

### 3.2. Transcription Factors

Transcription factors (TFs) regulate the complex transcription network and, therefore, are involved in a series of processes in alleviating effects of hypoxia and other abiotic stresses [37]. The differentially expressed TFs in this study belong to the top five gene families of MYB, bHLH, WRKY, NAC, and AP2-ERFs. Regulation of these gene families has been previously shown to respond to various stress factors including low temperature [41], salinity [42], and drought [43]. The striking downregulation of MYB family genes in hypoxic roots in our study may possibly be linked to root growth. *MYB83* was significantly downregulated in hypoxic roots, which might be linked with the collapse of cell walls [44]. NAC TFs have been reported to regulate cell death under multiple environmental stresses, and over 60 NAC genes from *B. napus* were previously identified [45]. It was reported that *NAC55* overexpression induces ROS accumulation and activates programmed cell death [46]. In our study, *NAC55* showed strong upregulation suggesting a possible link with oxidative injury after three days of hypoxia. However, the specific function of NAC55 still needs to be confirmed. RAP2.12, which belongs to AP2-ERF TFs, is the activator of hypoxic marker genes. Overexpression of *RAP2.12* enabled elevated levels of TCA cycle intermediates in *Arabidopsis*, which increased the plant survival rate via strongly upregulated hypoxia marker genes including *PDC1*, *ADH1*, and *SUS1* [47]. However, a similar gene, *RAP2.11*, in hypoxic *B. napus* roots displayed significant downregulation, which might explain the decrease of TCA cycle-related enzymes in our study. Concomitantly, *PDC1*, *ADH1,* and *SUS1* were sharply downregulated in hypoxic *B. napus* roots. A previous study suggested that AP2/ERF enabled direct activation of aquaporins including PIP2;2 and TIP1;1 in *Arabidopsis* [34], which might account for the downregulation of most of the aquaporins and, thereby, decreased *L*_pr_. The above responses may be partly responsible for the sensitivity of *B. napus* plants to hypoxia.

### 3.3. Ca^2+^ and Other Signaling Pathways

The involvement of Ca^2+^ in hypoxia responses has been reported for many plants, including muskmelon roots, in which Ca^2+^ was found to influence TCA cycles through regulating the ROS level [48]. An increase in Ca^2+^ concentration is detected by many Ca^2+^-sensing proteins (e.g., calmodulin (CaM), calmodulin-like proteins (CMLs), Ca^2+^-dependent protein kinases (CDPKs), and calcineurin B-like (CBL)/Ca^2+^-independent protein kinases (CIPKs)) [49]. In our study, the genes encoding these proteins, *BnCBL* and *BnCDPK12*, were downregulated after three days of hypoxia, pointing to a possible low Ca^2+^ concentration in cytosol and implicating the Ca^2+^ signaling pathway as a factor in the observed sensitivity of *B. napus* to hypoxia, which might explain the reason why the flooding tolerance of *B. napus* is limited compared with other species. In our study, the *NCED*, *ZEP* gene, which encodes key enzymes in the ABA biosynthesis pathway, was markedly upregulated by hypoxia. ABA was reported to participate in the production of ROS by affecting respiratory burst oxidase homologs (Rbohs). Collectively, these results indicate that *NCED*, *ZEP* [50] and other ABA generation-related genes may play regulatory roles in the ABA signaling pathway and hypoxia response in the roots of *B. napus*.

Ethylene initiates different adaptation strategies in plant species by regulating root growth and development under hypoxia stress, including adventitious root formation and root hair growth and development [51]. Ethylene is synthesized through the oxidation of ACC by ACC oxidase (ACO) [52]. In our study, most of the ACS- and ACO-related genes were downregulated suggesting a possible low ethylene entrapment in *B. napus* roots and a failure to activate hypoxia acclimation genes like *ADH* and *PDC* [19], likely contributing to the relative sensitivity of *B. napus* plants to flooding.

### 3.4. Transcriptional Responses Related to Cell Wall Modifications

Our data indicate that some of the cell wall-related genes play important roles in the acquisition of root hypoxia tolerance by changing their expression patterns. Plant cell walls play a structural role in plant abiotic stress in defending against pathogens [53]. It has been proposed that increasing the amount of suberin and lignin could efficiently delay plant cell damage by forming hydrated gels [54]. Interestingly, our data revealed upregulation of the gene expression of 1-acyl-sn-glycerol-3-phosphate acyltransferase (*GPAT1*, *GPAT3*), which is involved in the biosynthesis of suberin [55]. Our results also showed that the expansin proteins (*BnEXPB1*), which are responsible for wall loosening and reconstruction, were downregulated by three days of root hypoxia [56], suggesting a potential mechanism of cell wall modifications.

### 3.5. Transcriptional Responses Related to Root Water Relations

The marking reductions of physiological performance of hypoxic *B. napus* plants could be attributed to plant water relations. Specifically, the leaf water potential displayed a remarkable decrease by over three-fold compared with aerated plants. These results are consistent with previous studies in soybean [57] and tobacco [16], which showed downregulation of the aquaporins including most of the *PIP2*s and *TIP*s, the main water transporters in plant roots [40]. The massive downregulation of these genes may be associated with transcription factors including AP2-ERF [34].

### 3.6. Transcriptional Response Related to Redox Systems under Hypoxia Stress

The transcriptomic results demonstrated that genes contributing to ROS production and antioxidant enzymes were altered in *B. napus* by root hypoxia. The genes encoding antioxidant systems including SOD, APX, and POD, were mostly upregulated. Respiratory burst oxidase homologs (Rbohs) have been characterized as essential ROS-producing genes [46,58]. In our study, *RbohA*, *RbohB*, *RbohD*, and *RbohF* were markedly upregulated in response to three days of hypoxia in the distal root tissues. Root hypoxia commonly leads to ROS accumulation [58] and triggers antioxidant defense mechanisms [59]. In addition, ROS were reported to be associated with cell wall modifications through cleaving sugar bonds in polysaccharides of the cell walls [53]. Thus, our results demonstrate a fine-tuning of redox homeostasis, which may be the strategy of *B. napus* to survive under root hypoxia.

### 3.7. Transcriptional Responses Related to Respiration

Since the direct effect of hypoxia is the collapse of aerobic respiration processes, numerous genes encoding for the TCA enzymes and the genes involved in amino acid synthesis were downregulated in response to root hypoxia, which ultimately resulted in the energy crisis. Constitutive promoted fermentation is detrimental to plant growth, while conditional regulation of fermentation is essential for plant survival [60].

## 4. Materials and Methods

### 4.1. Plant Material and Treatments

Seeds of canola (*Brassica napus* L. cultivar Westar) were sterilized with 70% ethanol for 2 min followed by 1% (*v*/*v*) sodium hypochlorite for 30 min. The seeds were germinated at 20 °C on plates with half-strength Murashige and Skoog (MS) medium solidified with 0.8% agar. Shortly after germination, 60 seedlings were moved into sterilized plastic pots (one seedling per pot) filled with autoclaved peat moss/vermiculite (2:1 by volume). The seedlings were grown in a controlled-environment growth room with a 16 h photoperiod, 22/18 °C (day/night) temperature, 400 μmol m^−2^ s^−1^ photosynthetic photon flux density (PPFD), and 50–60% relative humidity. The seedlings were watered every other day and fertilized weekly with 50% modified Hoagland’s solution. Four-week-old plants were moved into the aerated hydroponic culture with half-strength Hogland’s nutrient solution in two 40 L plastic tubs (60 × 40 × 20 cm) and aerated to maintain a dissolved oxygen level of about 8 mg/L. After one week, half of plants from the two tubs were randomly selected and subjected to three days of hypoxia treatment in six tubs by flushing nitrogen gas through the nutrient solution to obtain an O_2_ concentration of about 2 mg/L. The remaining plants in another three tubs were aerated and served as control. For each measurement, plants were randomly taken from the different tubs.

### 4.2. Measurements of Gas Exchange, Leaf Water Potential, and Dry Weight

After three days of hypoxia treatment, net photosynthesis rate (*P*_n_), stomatal conductance (*g*_s_), and transpiration rate (*E*) were measured from three to six hours following the onset of the photoperiod using a Licor-6400 portable photosynthesis system with a 2  ×  3 cm^2^ leaf chamber (LI-COR, Lincoln, NB, USA). The reference CO_2_ concentration was set to 400 μmol mol^−1^, and the flow rate was 200 μmol s^−1^. The leaf chamber temperature was maintained at 20 °C, and the PPFD was 400 μmol m^−2^ s^−1^ of the red-blue light spectrum. Six to eight plants from each group were randomly selected and three fully expanded uppermost leaves from each plant were measured. The average values of the three leaves were used to calculate the means. After the measurements, the leaves were excised and immediately placed into a Scholander pressure chamber (PMS Instruments, Corvallis, OR, USA) for the measurements of leaf water potential (*Ψ*_leaf_) [61,62] (*n* = 6). Dry weights of plants were determined after one, three, eight, and twelve days of hypoxia in plant tissues dried in an oven at 80 °C for 72 h (*n* = 6).

### 4.3. Root Hydraulic Conductivity

After three days of hypoxia, root hydraulic conductivity (*L*_pr_) was measured using a hydrostatic method [62,63]. A 0.25 L glass cuvette containing half-strength Hoagland’s solution was inserted into a pressure chamber (PMS Instruments, Corvallis, OR, USA). Roots were excised above the root collar and sealed in the pressure chamber. The whole root system was immersed in the solution. The measurements were carried out by determining the volume of exuded sap after pressurizing the chamber to 0.3, 0.4, 0.5, and 0.6 MPa for 2 min at each pressure to establish root water flow rates [62]. Root hydraulic conductance (*K*_r_) was determined from the slopes of the pressure/volume relationship [4]. Root hydraulic conductivity (*L*_pr_) was obtained by dividing the *K*_r_ by the root volume. The light green SF yellowish dye (250 μmol L^−1^, Sigma-Aldrich Chemical, Oakville, ON, Canada) was used to determine the relative contribution of apoplastic pathway to *K*_r_ and 400 mM AgNO_3_ as an aquaporin water transport inhibitor.

### 4.4. Anatomy of Distal Root Parts

Thin sections of distal root segments (*n*  =  4–6) from each treatment were prepared for microscopy [64]. Distal parts of roots, 3–5 cm in length, were fixed in formalin-acetic acid-alcohol (FAA) solution. After fixation, the root segments were dehydrated in an ethanol series and placed in toluene. Fixed root segments were embedded in paraffin and sectioned with a microtome. Lignin autofluorescence was visualized following UV excitation at 330 nm to 380 nm with a fluorescent microscope (Carl Zeiss, Jena, Germany) [65]. For suberin visualization, thin paraffin sections were incubated for 30 min at room temperature in a freshly prepared solution of 0.01% Sudan 7B in lactic acid (*w*/*v*) and then rinsed in 90% ethanol (two baths of 1 min each) [66]. The sections were then mounted on slides in 50% glycerol and observed under a compound light microscope (ZEISS AXIO, Carl Zeiss, Jena, Germany).

### 4.5. Transcriptome Sequencing

Total RNA of distal roots (3–5 cm) of three biological replicates was collected, immediately frozen in liquid nitrogen, and stored at −80 °C. RNA was extracted using the RNeasy Plant Mini Kit (Qiagen, Hilden, Germany). The RNA quality was determined via gel electrophoresis, and the RNA concentration was determined using a NanoDrop spectrophotometer (Thermo Fisher Scientific Inc., Waltham, MA, USA). The integrity of six RNA samples (three hypoxia treatment and three control samples) was determined with Agilent 2100 BioAnalyzer^®^ (Agilent Technologies™, Santa Clara, CA, USA), and all three samples had RNA integrity number (RIN) values higher than 8.5. Purification of the poly-A containing mRNA molecules was performed, and the mRNA was fragmented followed by synthesis of the second strand cDNA. The products were then purified and enriched with PCR amplification. Illumina sequencing of each cDNA library was performed using the GAII platform at the Beijing Genomics Institute (BGI-Shenzhen, Shenzhen, China http://www.genomics.cn/en/index, accessed on 9 July 2010.) according to the manufacturer’s instructions (Illumina, San Diego, CA, USA). The library was then sequenced using an Illumina HiSeq 4000 platform (BGI-Shenzhen, China) (reads length 151 bp). Sequenced reads were deposited in the NCBI Sequence Read Archive (SRA) with the accession number PRJNA637154.

### 4.6. Data Processing and Analysis

The RNA-seq reads generated by the Illumina Genome Analyzer and sequencing reads filtering was processed with SOAPnuke Software [67]. Low quality reads, reads with adaptors, and unknown bases were removed. Afterwards, the clean reads were used for downstream bioinformatics analysis. In the pipeline, clean reads were mapped on the *B. napus* reference sequence (https://www.ncbi.nlm.nih.gov/assembly/GCF_000686985.2, accessed on 15 September 2017) using HISAT2 tools (v2.0.4) [68]. RSEM software was used to calculate the gene expression value (FPKM, fragments per kilobase of transcript length per million mapped reads). DESeq2 software was used to identify differentially expressed genes (DEGs). Genes with a false discovery rate (FDR) ≤ 0.05 and log2 fold-change (FC) ≥ 2 found using DESeq2 were considered to be differentially expressed genes.

### 4.7. KEGG, GO Enrichment, and Pathway Analysis

Gene function was annotated based on the KEGG (Kyoto Encyclopedia of Genes and Genomes) and GO (Gene Ontology) database. The pathway enrichment analysis of DEGs was implemented using the phyper R package. FDR ≤ 0.01 were considered to be significantly enriched.

### 4.8. qRT-PCR Validation

For the qRT-PCR analysis, the distal root samples of control and hypoxia were collected after three days of hypoxia treatment for the extraction of RNA. The experiments were performed with three biological replicates, and three technical replicates were included. The samples were quickly frozen and kept in liquid nitrogen before being transferred to the –80 °C freezer. The samples were ground with a mortar and pestle in liquid nitrogen. Total RNA was extracted with an RNeasy Plant Mini Kit (Qiagen, Hilden, Germany). First-strand complementary DNA (cDNA) synthesis and qRT-PCR were conducted as described [69]. Expression data were calculated with the Livak calculation method and presented as log (2^−ΔΔCt^) [70]. Relative transcript fold change was normalized against the geometric mean of the CT value of two reference genes, *BnACTIN7* (AF_111812) and *BnPPR* (XM_013831783). The primers used in this study are listed in Supplementary Material Table S1.

### 4.9. Data Analysis

Each measurement included 4–6 independent biological replicates and the significance level was set at *p* ≤ 0.05. The data were analyzed via an independent samples *T*-test using Excel (Microsoft, Redmond, WA, USA). The differences between gene expression levels were analyzed via one-way ANOVA (Tukey’s test; *p* ≤ 0.05). A heatmap of TFs and the schematic pathway was constructed based on the transcriptome data using the pheatmap package with R software (http://www.r-project.org/, accessed on 10 March 2022).

## 5. Conclusions

**Figure 10:**
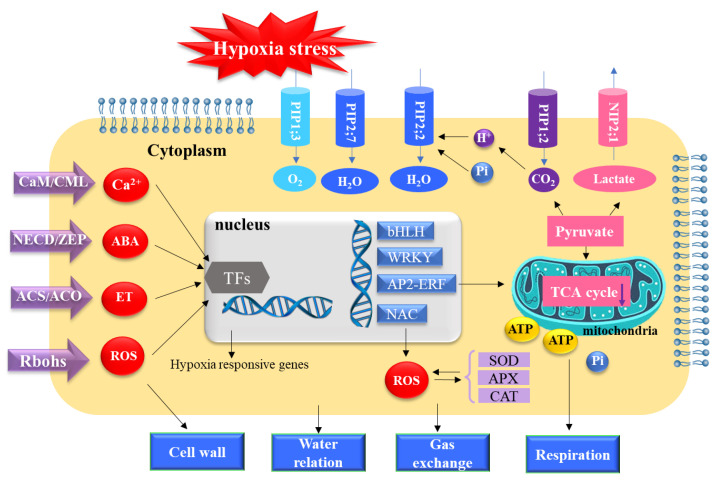
A model based on transcriptomic analysis for the responses of distal root tissues in canola to three days of root hypoxia. Hypoxia stress triggers alterations of cytosolic Ca^2+^, ABA, ethylene, and reactive oxygen species (ROS). Hypoxia-responsive signaling substances activated expression alteration of TF regulators including bHLH, AP2-ERF, WRKY, and NAC. The AP2-ERF expression was linked to downregulation of the TCA cycle, which resulted in ATP depletion leading to the energy crisis and affecting gas exchange and protein phosphorylation. As aerobic respiration was inhibited under hypoxia stress, fermentation activated NIP2;1 and PIP1;2 and helped with the efflux of lactic acid and CO_2_ respectively. The NAC TFs were associated with generation of ROS, and ROS activated antioxidant systems involving SOD, APX, and POD. *PIP1*;*3* was upregulated, possibly to enhance the oxygen influx into the cytoplasm. The *PIP2*;*7* was upregulated to keep water homeostasis. Decreased pH and phosphorylation led to closure of water-transporting aquaporins, including *PIP2*;*2*, and led to decreases in *L*_pr_ and, consequently, *Ψ*_leaf_. Increased ROS were associated with the cell wall modifications of root distal segments, gas exchange, and water relations of hypoxic canola plants.

Differential expression analysis identified genes in the tissues of canola distal root regions that responded to the root hypoxia treatment. Profound differences were found between the transcriptomes of control and hypoxic plants. We propose a model, which describes the limited hypoxia tolerance mechanisms in waterlogging-sensitive plants based on the results of physiological responses and transcriptomics (Figure 10). The genes involved in the ABA, ROS, ethylene, and calcium signaling pathways were altered via hypoxia. Most of the decreased transcripts were related to *L*_pr_ and respiration. The data demonstrated high sensitivity of canola to root hypoxia. The alteration of TFs expression suggested defense pathways in the distal root zone. The results fundamentally advance the understanding of how modified gene expression patterns facilitate acclimation to root hypoxia stress.

## Figures and Tables

**Table 1:** Summary of RNA-Seq performed for *Brassica napus* root distal segments after three days of hypoxia.

Sample	Total Reads	Total Mapped Reads	Perfect Match	Unique Match	Multi-Position Match	Total Mapping Ratio
Aerated 1	46,775,054	34,595,274	21,507,365	8,113,278	26,481,996	77.59%
Aerated2	49,481,950	38,117,812	23,547,536	8,056,774	30,061,038	78.58%
Aerated 3	49,581,202	36,739,522	22,957,266	7,792,122	28,947,400	75.36%
Hypoxia 1	49,309,128	36,636,548	22,409,722	8,292,208	28,344,340	78.08%
Hypoxia 2	49,442,460	36,211,850	22,619,563	8,073,762	28,138,088	77.49%
Hypoxia 3	45,939,022	33,218,102	20,689,475	7,563,604	25,654,498	76.80%
Average	48,421,469	35,919,851	22,288,488	7,981,958	27,937,893	77.32%

## Data Availability

All data supporting the findings of this study are available within the paper and its Supplementary Information files.

## Supplementary Materials

The following supporting information can be downloaded at: https://www.mdpi.com/article/10.3390/ijms232415469/s1, Table S1: Primer sequence for qRT-PCR analysis.
