# Supplementary material for: Transcriptomic Analysis of Distal Parts of Roots Reveals Potentially Important Mechanisms Contributing to Limited Flooding Tolerance of Canola (Brassica napus) Plants
Source: Int J Mol Sci. 2022 Dec 7;23(24):15469. doi: 10.3390/ijms232415469 (PMC9779561; doi:10.3390/ijms232415469)
Supplement: Supplementary file 1 [file ijms-23-15469-s001.zip › ijms-2068330-supplementary.pdf]

TableS1.Primer sequence for qRT-PCR analysis

Gene name	accession number	Forward primer(5`-3`)	Reverse primer(5`-3`)
BnPIP1;1	XM_009118558.3	5'-GCTAAGAACAGACCAAAAGTG-3′	5'-GGCATTCGGTGGTATGATA-3′
BnPIP1;2	XM_013892758.2	5'-GAATGGTCCAACCCAAAAG-3′	5'-TGTTCCCATTCTTGCACCT-3′
BnPIP1;3	XM_009120575.3	5'-CTGGAAGCCTTTGACAACG-3′	5'-CATAAACCCAGCGGTGACA-3′
BnPIP2;2	XM_013836821.2	5'-GGAGTCTCGGGCATTTCTT-3′	5'-CGATGGATACAGCACAGGG-3′
BnPIP2;5	XM_009105687.3	5'-GTGTAGTAAGAAGACTGGAAGGA-3′	5'-GAAAGTGACGTTGGTGAGG-3′
BnPIP2;7	XM_013815142.2	5'-TGGGATGGTAGCCAAATGC-3′	5'-TTCTCCGCGACTGACCCT-3′
BnTIP1;1	XM_009143362.3	5'-TTTTTGTCTTTTGCGTTTGTG-3′	5'-GGAAATCAACAGAAAAGGATTCA-3′
BnNIP5;1	XM_013880108.2	5'-ACGTCCCAGATCCATTTTCTT-3′	5'-CCAGGACTAGTTTCACCAAACAG-3′
BnNCED	XM_013732216.1	5'-GTCGCGTCAACCTCCAAGCT-3′	5'-TCTGTTTCTCCCCGGAGAGG-3′
BnNAC55	XM_009124259.3	5'-CACTCGCACCGCCTCAAAAC	5'-CCCATGGACGCAGAAAAGAATAAG-3′
BnERF2	XM_033280981.1	5'-GGGTTTGGTTAGGGACGTTTG	5'-GGAACCACGCATCCTAAAAGC-3′
BnRbohF	XM_013825233.2	5′ AGAACGTTGAAGGGTGGAAC 3′	5'-AGCTCCAATGCAAACTCCTT-3′
BnRbohD	XM_013788801.2	5'-TGTAGAGCCGTCTCTCCCTT-3′	5'-ACGGTCCTGAGCTTACGAGT-3′
BnMYB108	NM_001315555.1	5'-GCCTCCGCCGCATCATTA-3′	5'-GTTCGGTTTTGGAGCGTTGTAGTA-3′
BnWRKY40	XM_013732543.1	5'-CATTCACTCGTTTTAATTCGTCCAC-3′	5'-CGCGCATATACGTACATACTCACAA-3′
BnADH	XM_013832947.2	5'-CTAAGTTCATCATGTCTACGACCGG-3′	5'-TCGGTGTGACAGAGAGAAGTGAAG-3′
BnLDH	XM_013886552.2	5'-ATTGTGTAATGGCGAACAACGG-3′	5'-ATGACGGAGACCTTGGTGCA-3′
BnPDC	XM_013852099.2	5'-TGGAGGGTACACCATTGAAGTGG-3′	5'-CGCAGTCCAGCATTTACCTTCTC-3′
BnGAPDH	XM_013801897.2	5'-TTAGACTCGAGAAAGCTGCGACC-3′	5'-CTGTTGTCACCAACGAAGTCAGTTG-3′
BnGLB	XM_009124578.3	5'-ATTGGTGAGGACACTGAAAGAA-3′	5'-TCTCGGCCTTAATGGCTAAAG-3′
BnCPK12	XM_013869200.2	5'-AGTGGCTGCATTCTCTTTCTTTGAC-3′	5'-ATCCCTCCACTCCCATTTCCTT-3′
BnCPK11	XM_009146872.3	5'-TGGTGGCAGCATTTTCGTATTTT-3′	5'-TTCTCCCAACTCCATCTCCTTTCTT-3′
BnPPR	XM_013831783	5'-TGGTGTGCGATAAGTGTGAGA-3′	5'-GGTGTCCATCTGTTCTTCTTGG-3′
Bnactin7	XM_013858992.2	5'-CCCTGGAATTGCTGACCGTA-3′	5'-TGGAAAGTGCTGAGGGATGC-3′
